# Isotherm and kinetic studies of cadmium biosorption and its adsorption behaviour in multi-metals solution using dead and immobilized archaeal cells

**DOI:** 10.1038/s41598-023-29456-5

**Published:** 2023-02-13

**Authors:** Ghada E. Hegazy, Nadia A. Soliman, Mona E. Ossman, Yasser R. Abdel-Fattah, Madelyn N. Moawad

**Affiliations:** 1 National Institute of Oceanography and Fisheries, NIOF, Cairo, Egypt; 2 Bioprocess Development Department, Genetic Engineering and Biotechnology Research Institute (GEBRI), City of Scientific Research and Technological Applications (SRTA-City), New Borg Elarab City, Alexandria Egypt; 3 Environment and Natural Material Research Institute (ENMRI), City for Scientific Research and Technological Applications (SRTA-City), New Borg Elarab City, Alexandria Egypt

**Keywords:** Biological techniques, Biotechnology, Environmental sciences

## Abstract

It is crucial to identify more biological adsorbents that can efficiently uptake metals from wastewater. Dry haloalkaliphilic archaea *Natronolimnobius innermongolicus*was evaluated for Cd ions biosorption. The optimal operating conditions (pH, biomass dose, initial metal concentration, contact time, and isotherms models) were tested. Biosorption process is influenced by the metal's solution pH with maximum removal of 83.36% being achieved at pH 8. Cadmium ions uptake reaches equilibrium in about 5 min of biosorption process. The Langmuir model was determined to better fit the Cd(II) biosorption by dry archaea. The maximal uptake capacity (q_max_) of Cd(II) was 128.21 mg/g. The effect of multi-component system on biosorption behaviour of Pb, Ni, Cu, Fe, and Cd ions by immobilized dried archaeal cells, dried archaeal cells, and dried bryozoa was studied using Plackett–Burman experimental design. The investigated biosorbents were effective at removing metals from contaminated systems, particularly for Fe, Pb, and Cd ions. Moreover, the interaction behaviour of these metals was antagonistic, synergistic, or non-interactive in multi-metals system. SEM, EDX, and FTIR spectra revealed changes in surface morphology of the biomass through the biosorption process. Finally, continuous adsorption experiment was done to examine the ability of immobilized biomass to adsorb metals from wastewater.

## Introduction

The presence of heavy metals in marine environments is recognized to cause aquatic life damage^1, 2^, because these harmful heavy metals kill microorganisms during wastewater treatment which lead to a delay in the purification process of water^3^. Heavy metals are resulted from many industries such as pigments, textile, petroleum refining, plating and battery manufacturing. Also other contamination sources of heavy metals include agricultural chemicals such as fertilizers and pesticides^4^. The traditional methods to eliminate the heavy metals from contaminated aqueous solutions involve filtration, ion exchange, chemical oxidation or reduction, chemical precipitation, recovery using evaporation, and electrochemical treatment^5, 6^. However, these traditional methods are cheap but they are ineffective when the metal concentration in the contaminated water is less than 100 mg/l^7^, so in this case, the biosorption process can be more effective than the traditional methods at low metal concentration and also have the benefits of a low operating expense^8, 9^. Biosorption process is defined as "the ability of biological materials to remove toxic heavy metals from contaminated aqueous environments through physicochemical reactions or metabolically mediated of uptake, or by binding and concentrating heavy metals by non-living and inactive biological biomass from contaminated aqueous solutions by absorbing toxic ions of heavy metals on the outer cellular cell wall (the surface adsorption mechanism)"^1, 10^. Fungi^11^, algae^12–17^, and bacteria^18^ have been well-known to adsorb metal ions. To improve the biosorption process in wastewater treatment, it is important to recognize more new biological biomass that could uptake metals from the aqueous solutions with high efficiency and to design bioprocesses that efficiently remove heavy metals from polluted marine environments.

Extreme microorganisms, especially archaea have the ability to survive in extreme environments with harsh conditions such as high acidity, high temperature, high pressure, anoxic conditions, and the absence of organic matter^19^. This extremophilic archaea cell wall consists of a glycoprotein surface layer (pseudo-crystalline proteinaceous surface layer). These proteins of S-layer can protect archaeal cells from extreme hard conditions and preserve the shape of archaeal cell (exoskeleton). Moreover, archaeal protein glycosylation have very important role in S-layer thermal stability^19^. Extremely halophilic archaea have been successfully isolated from salt soda lake at Wadi El-Natrun. They can exist at high salt concentrations up to 4 M, pH values as high as 11, and temperatures ranging from 37 to 50 °C. They can also grow anaerobically and aerobically^20^. Taking *Natronolimnobiusi nnermongolicus* strain GHWN83as a model for haloalkaliphilic archaea in the present study, it was immobilized with the marine invertebrate bryozoan *Bugula neritina* to boost the ability of its surface to adsorb heavy metals, where this *Cheilostome bryozoa* develops its skeleton from calcite and aragonite^21^. The development of new and novel, cost effective and efficient immobilized mass for heavy metals polluted water treatment is actually limited and expensive so in this study, we will use novel combined immobilizedbiomass material (haloalkaliphilic archaea with the marine invertebrate bryozoan *Bugula neritina*). Also we aim to investigate the optimal operating conditions (e.g. pH, biomass dose, initial metal concentration, contact time, and isotherms models) for cadmium biosorption using dried GHWN83 strain of *Natronolimnobius innermongolicus*, a marine halophilic archaeon isolated from the Egyptian lake (El-Hamra lake), Wadi El-Natrunkept in Gene Bank (ac: OM302165). Plackett–Burman experimental design was conducted to evaluate the effect of multi-component system on the biosorption capacity of immobilized dried archaeal cells, dried archaeal cells, and dried Bryozoa biomass. The surface characterization of biosorbents was also investigated during biosorption process. Finally, the application of the biosorption process to wastewater was completed using the continuous adsorption experiment.

## Materials and methods

### Biomass preparation

#### Archaeal isolation and identification

Water and sediments samples were collected from the Egyptian lake Wadi El-Natrun (salinity reach to more than 4 M NaCl, pH value reach 11 and temperature up to 50 °C). The culture medium used in this study was prepared using distilled water. The pH was adjusted to 11 using Na_2_CO_3_, and the medium was sterilized by autoclaving at 120 °C for 20 min. The following were in g/l constituted the isolation basal medium: Casamino acids, 5; KH_2_PO_4_, 1; MgSO_4_.7H_2_O, 0.2; NaCl, 200; trace metals, 1 ml; and Na_2_CO_3_, 18. The trace metal mixture included (g/l) ZnSO_4_.7H_2_O, 0.1; MnCl_2_.4H_2_O,0.03; H_3_BO_3_, 0.3; CoCl_2_.6H_2_O, 0.2; CuCl_2_.2H_2_O, 0.01; NiCl_2_.6H_2_O, 0.02; and Na_2_MoO_4_·H_2_O, 0.0326. The purification process led to the isolation of an extreme haloalkaliphilic archaeon coded as GHWN83 from El-Hamra Lake, Wadi El-Natrun, Egypt. For molecular identification, a simplified rapid protocol was used for the preparation of archaeal DNA. A polymerase chain reaction (PCR) was carried out to amplify the 16S rRNA genes from archaeal genomes using universal primers designed to amplify ~ 1500 bp of this gene, which were then sequenced. To evaluate similarity, the BLAST tool was utilised. Thereafter, the acquired sequence was added to the Gene Bank.

#### Archaeal biomass preparation for biosorption study

Archaeal culture grown for 7 days until the logarithmic phase (OD_600nm_ of 0.99) was filtrated, autoclaved and then dried in oven at 60 °C overnight. For next experiments, the dried biomass was utilized. The pH_PZC_ of dried archaea was measured according to the method described by^22^. The initial pH values were plotted against ΔpH to obtain pH_PZC_, the point where the pH_initial_ equal to pH_final_.

#### Preparation of immobilized biomass

Dry bryozoa species, *Bugula neritina* was used as solid support. The dry archaeal biomass was introduced to the dry *B. neritina*. Solid support with immobilized dry archaeal cells (IABB) was washed with distilled water and dried for overnight in oven at 60 °C. The biosorption capacity of immobilized biomass was tested under experimental conditions of 50 mg/l initial Cd(II) concentration, temperature 25 °C, and pH 8 and compared with metal uptake capacities of the dry archaea without solid support (AB) and bryozoa without archaea (BB).

### Reagents and equipment

Deionized water was used throughout the experiments. Individual stock metals solutions of 750 mg/l from Cd, Pb, Ni, Cu, and Fe ions were made from CdCl_2_·H_2_O, Pb(NO_3_)_2_, NiCl_2_.6H_2_O, CuSO_4_.5H_2_O, and FeCl_3_ salts. The required concentrations were prepared by adequate dilution of stock solution with deionised water. The initial pH was adjusted using 0.1 M of HCl or NaOH. All chemicals were of analytical grade (Merck, Mumbai, India). Metal concentrations were measured using atomic absorption spectroscopy (AAS Analytical Jena AG, 07745 Jena, Germany). Samples were diluted prior to analysis to set within the calibration linear range.

### Biosorption studies

#### Determination of metal removal efficiency

The metal solutions after the biosorption process were collected, filtered, and subjected to AAS analysis for the analysis of metal ions in order to assess the removal rate of metal ions by biosorbents. Biosorption capacity (q_e_), the amount of metal adsorbed per gram of biosorbent, can be calculated at equilibrium in mg/g as follows:

1 $${\text{q}}_{{\text{e}}} = \left( {{\text{C}}_{{\text{o}}} - {\text{C}}_{{\text{e}}} } \right){\text{V}}/{\text{m}}$$

where C_o_ is the initial concentration of metal ions in the solution (mg/l), C_e_ is the equilibrium concentration of metal ions in the solution (mg/l), V is the volume of solution (in litres), and m is the mass of biosorbent applied, in grams. Metal uptake can also be expressed as percentage of metal removal, given by:

2 $${\text{Metal}}\,{\text{removal}}\,\left( \% \right) = {1}00\left( {{\text{C}}_{{\text{o}}} - {\text{C}}_{{\text{e}}} } \right)/{\text{C}}_{{\text{o}}}$$

#### Factors influencing biosorption

The impact of various operational factors on biosorptionsuch as pH values (2. 4, 6, 8, and 10), dry archaeal cells dose (0.5, 1, 1.5, 5, 3, and 4 g/l), initial heavy metal concentration (10, 25, 50, 100, 150, 250 mg/l), and contact time (0, 1, 2, 5, 10, 20, and 30 min) was explored for Cd(II) uptake. While retaining the other experimental parameters constant, the parameter under study was altered.

#### Kinetic study

At pH 8, 1 L of Cd ion solution (50 mg/l) was added to 0.5 g of biosorbent. The solution was stirred at constant temperature (25 °C) and speed (150 rpm). Samples were collected at intervals of 0, 1, 2, 5, 10, 20, and 30 min, filtered, and subjected to an AAS analysis. The adsorption kinetics of Cd(II) using dry archaea was analysed using pseudo-first-and pseudo-second-orders^23^. The pseudo-first-order is given by:

3 $${\text{log}}\left( {{\text{q}}_{{\text{e}}} - {\text{q}}_{{\text{t}}} } \right) = {\text{log q}}_{{\text{e}}} {-}\left( {{\text{k}}_{{1}} /{2}.{3}0{3}} \right){\text{t}}$$

where q_e_ and q_t_ (mg/g) are the adsorption capacities at equilibrium and at time t, respectively. K_1_ (min^−1^) is the rate constant of pseudo-first-order adsorption. When values of log (q_e_ − q_t_) are linearly linked with t, a good match for the pseudo first-order is obtained. Based on the kinetic model, the values of k_1_ and q_e_ are determined from the slope and intercept, respectively. The pseudo-second-order is given by:

4 $${\text{t}}/{\text{qt}} = {1}/{\text{k}}_{{2}} {\text{q}}^{{2}}_{{\text{e}}} + {\text{t}}/{\text{q}}_{{\text{e}}}$$

where k_2_ (g/mg min) is the pseudo-second order rate constant of sorption. The plots of t/qt against t should give a linear relationship from which the value of q_e_ and k_2_ can be determined from the slope and intercept, respectively.

#### Biosorption isotherms

10 ml of Cd(II) solutions with various metal concentrations (10–250 mg/l) were combined with 5 mg of biosorbent at pH 8. For 1 h, a temperature of 25 °C and stirring of 150 rpm were maintained for the solution. Experimental adsorption data were described by the two most used isotherms, the Langmuir and Freundlich models^24, 25^. The Langmuir isotherm assumes monolayer coverage of metal ions over a homogenous sorbent surface, the adsorption of each molecule on the surface has equal adsorption activation energy, each site can occupy only one molecule or atom and in close proximity, adsorbed molecules or atoms do not interact with one another. The isotherm is presented by the following equation:

5 $${1}/{\text{q}}_{{\text{e}}} = {1}/\left( {{\text{bq}}_{{{\text{max}}}} {\text{C}}_{{\text{e}}} } \right) + {1}/{\text{q}}_{{{\text{max}}}}$$

where q_e_ (mg/g) is the observed biosorption capacity at equilibrium, q_max_ (mg/g) is the maximum biosorption capacity corresponding to the saturation capacity (represent total binding sites of biomass), C_e_ (mg/l) is the equilibrium concentration, and b (l/mg) is the energy of adsorption. The Freundlich isotherm implies a heterogeneous surface, non-uniform heat of adsorption distribution throughout the surface (binding sites are not equal), and multi-layered adsorption. The mono-component Freundlich isotherm equation is given by:

6 $${\text{logq}}_{{\text{e}}} = {\text{logk}}_{{\text{f}}} + {1}/{\text{n}}\left( {{\text{logC}}_{{\text{e}}} } \right)$$

where k_f_isFreundlich isotherm constant and n is the constant related to the affinity of metal ions on the adsorbent. By plotting log q_e_Vs log C_e_, the constant n and k_f_ can be determined from the slope and intercept, respectively.

### Plackett–Burman batch experiment

Plackett–Burman experimental design was used to evaluate the relative significance of 5 factors^26^. Based on the Plackett–Burman factorial design, each factor was examined at 2 levels: ʻ − 1ʼ for the low level, and ʻ + 1ʼ for the high level. In addition, the matrix design of the tested factors was screened in 8 experimental trials. All trials were done on IABB, AB, and BB to detect the effect of different variables on the biosorption process either positively or negatively. The studied 5 variables were Pb, Ni, Cu, Fe, and Cd metals ions. At a temperature of 25 °C, biomass was mixed with the metals solution for 1 h. Plackett–Burman design is based on a first order model:

7 $${\text{Y}} =\upbeta _{0} + \sum\upbeta _{{\text{i}}} {\text{x}}_{{\text{i}}}$$

### Biosorbent characterization throughout biosorption study

#### Scanning electron microscopy and energy-dispersive X-ray spectroscopy (EDX) analysis

The differences in surface morphology of biosorbents were examined using emission scanning electron microscope (SEM, JEOL JSM 5500 LV) equipped with energy-dispersive X-ray spectroscopy (EDX) before and after biosorption process under ambient conditions (Cd(II) concentration (50 mg/l), temperature (30 °C), and pH 7). The IABB samples were first coated with gold to avoid charging and were determined at magnification of 80–5000×. EDX was used to determine the elemental composition of IABB.

#### Fourier transform infrared spectroscopy (FT-IR)

Alterations in functional groups on IABB were determined throughout the biosorption approach (raw and metal-loaded biomass). The samples were mixed with KBr and the FTIR spectra were analyzed using a model Perkin–Elmer IR spectrometer within the wavenumber 400–4000 cm^−1^ under ambient conditions.

### The continuous adsorption studies (Continuous Fixed-Bed Column)

**Figure 1:**
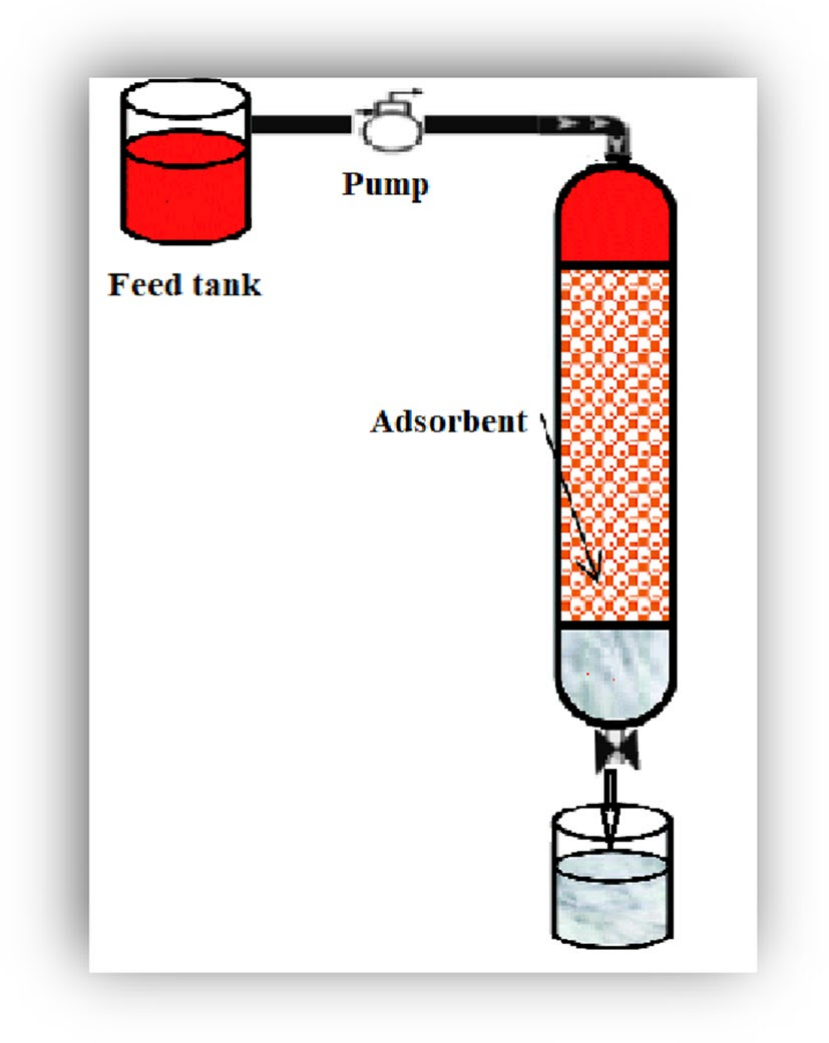
The continuous adsorption experiment.

The continuous adsorption experiment for wastewater treatment took place using the experimental device manufactured from rigid PVC transparent tube with internal diameter of 2.5 cm and 40 cm height (Fig. 1). The adsorptive material was packed into the column and humid with pure water to bring out the cornered air between the particles. Within the bottom, a screen of 0.05 cm thickness pendent with glass wool was used to forestall any loss of adsorbent and to form a decent support to the adsorbent bed. Wastewater was continually provided through the column with a flowrate of 6 ml/min. Grab samples were collected each ten minutes from the bottom of the column and were tested to recognize the concentration of the heavy metals under study. The widely used models for study the column behaviour are the Thomas and Yoon–Nelson.

#### Thomas model

Thomas model is used to determine the Thomas rate constant (KTH) and maximum adsorption capacity (q_e_) for column data through Eq. (8) by plotting C_0_/C_t_ against t^27^.

8 $${\text{ln}}\left[ {\left( {{\text{C}}_{0} /{\text{C}}_{{\text{t}}} } \right) - {1}\left] = \right[\left( {{\text{KTHq}}_{{\text{e}}} {\text{x}}/{\text{Q}}} \right) - {\text{KTH C}}_{0} {\text{t}}} \right]$$

where C_0_ is the initial concentration (mg/l), C_t_ is the outlet concentration in time t (mg/l), KTH is Thomas rate constant (L min^−1^ mg^−1^), q_e_ is the equilibrium adsorption capacity (mg/g), x is the mass of adsorbent in the column (g), and t is the time (min).

#### Yoon–Nelson model

Yoon–Nelson model was used to determine the Yoon–Nelson rate constant (KYN) and the time required for 50% adsorbate breakthrough (τ) for column data through Eq. (9) by plotting ln (C_t_/C_0_ − C_t_) against t^28^.

9 $${\text{ln}}\left( {{\text{C}}_{{\text{t}}} /{\text{C}}_{0} - {\text{C}}_{{\text{t}}} } \right) = {\text{KYNt}}{-}\tau {\text{KYN}}$$

where C_0_ is the initial concentration (mg/l), C_t_ is the outlet concentration in time t (mg/l), Cis Yoon–Nelson rate constant rate constant (min^−1^), t is the time (min) and the τ is the time required for 50% adsorbate breakthrough (min).

## Results and discussion

### Biosorption properties of Cd ions using dry archaeal biomass (AB)

#### The pH effect and the point of zero charge (pHpzc)

**Figure 2:**
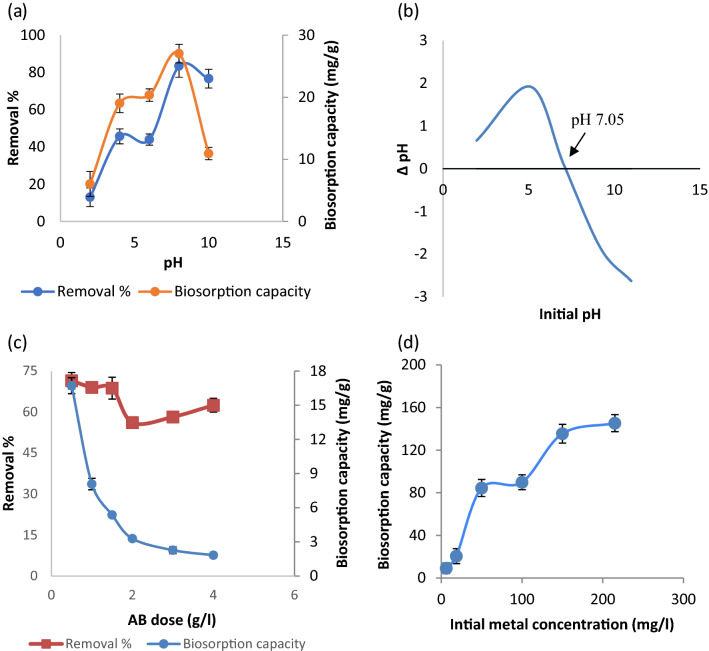
Biosorption properties of Cd ions using dry archaeal biomass (AB); (**a**) effect of pH, (**b**) isoelectric point of AB, (**c**) AB dose, (**d**) initial metal concentrations.

The cadmium uptake by thermally inactivated biomass was investigated at different pH and the results were shown in Fig. 2a. The maximum removal percentage was 83.36% at pH 8, with 20 mg/l of initial Cd(II) concentration and 2 g/l of biosorbent dose at room temperature. At low pH (< 6) and high pH (> 10) range, the cadmium uptake was dropped down being 43.95% and 76.61%, respectively. The biosorption process is influenced by the metal's solution pH, which results in changes to the functional groups on the biosorbent surface and to the metal's solution chemistry^22^. The biomass pH_PZC_ might describe the alterations in biomass surface. The pH of the solution at which the overall charge on the biomass surface is zero, is defined as pH_PZC_^29^. In the present study, the pH_PZC_ value was 7.05 for archaeal biomass (Fig. 2b). If the pH of the metal solution falls below pH_pzc_ (7.05), the cell surface becomes less negatively charged, limiting metal biosorption capability of biomass. When the pH value of metal solution exceeds pH_PZC_ (7.05), deprotonation of binding sites proceeds, rendering negatively charged functional groups on the cell wall accessible for metal binding^29–31^. However, the uptake of Cd ions declined beyond pH 8.0. At higher pH (> 8), the generation of soluble hydroxylated metal ion complexes may diminish biosorption ability that compete with metal ions for active sites^32^ leading to the reduction in metal ions retention on biomass. As a result, further biosorption studies were carried out at pH 8.0. Similar maximum Cd(II) removal at pH (8.0) was reported for filamentous fungi^33^ and for *Ochrobactrum anthropi*^34^. On the other hand, the optimal pH of 6 for Cd(II) adsorption was recorded for *Pantoea agglomerans* UCP1320^35^, *Escherichia coli*, *Bacillus subtitis*^36^, *Pantoea* sp.^37^, and *Pseudomonas aeruginosa* PU21^38^. Hou et al.^39^ reported that cadmium biosorption by *Klebsiella* sp. was optimal at pH 5.0.

#### Biosorbent dose

Regarding metal removal (%), the increase in biomass dose from 0.5 to 4.0 g/l reduced metals removal relatively from 71.48 to 62.51% for Cd(II) (Fig. 2c). The sharp reduction in biosorption capacity was observed with increasing biomass dose. The values dropped from 16.72 to 1.83 mg/g as the biomass dosage increased (0.5–4.0 g/l). A similar pattern has been described with variation in biosorbent dosage for Cd(II)^22, 30^. At high biosorbent dose, cell aggregation occurs causing a reduction in intercellular distance and the protection of binding sites from metal ions^30^.

#### Initial cadmium concentration

The biosorption capacity of Cd(II) rose with increasing initial concentration up to 215 mg/l with biosorption capacities of 145.35 mg/g (Fig. 2d). So the minimum and maximum metal sorptions of 9.16 mg/g and 145.35 mg/g occurred at Cd(II) concentration of 6 and 215 mg/l, respectively. Karakagh et al.^40^ observed similar results for Cd(II) removal using *Actinomyces* sp., *Streptomyces* sp., and *Bacillus* sp. The differences between the concentration of metal ions on the cell surface and in the bulk solution created a strong thrusting force that facilitated the biosorption process^30, 41^. However, with increasing metal concentrations (> 150 mg/g), the rate of uptake capacity tends to decline, resulting in near-constant saturation values. This reflects a shortage in binding sites^42^.

#### Isotherm study

**Figure 3:**
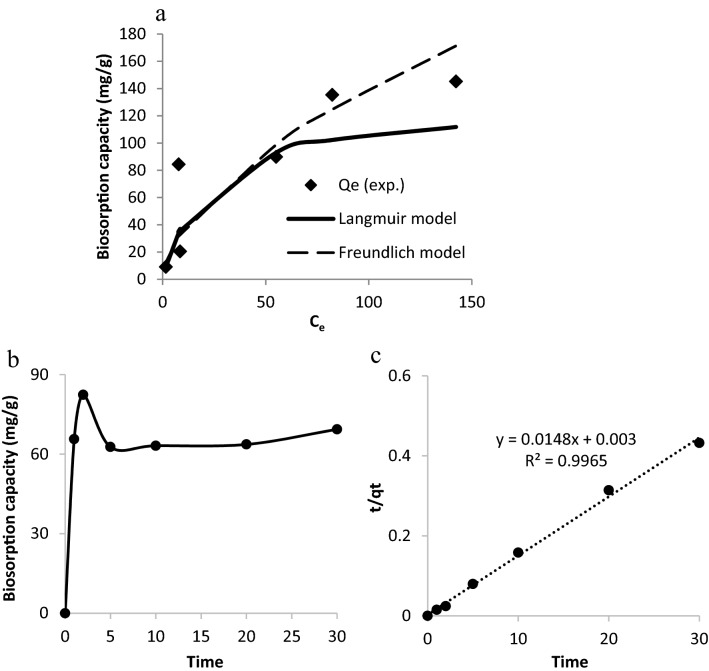
Isotherm and KINETIC studies of Cd ions removal by dry archaeal biomass AB; (**a**) comparison between different isotherm models and experimental data, (**b**) effect of contact time, (**c**) second-order equation.

The metal distribution between wastewater and biosorbent was depicted using the Langmuir and Freundlich models (Fig. 3a)^24, 25^. The Langmuir and Freundlich isotherms for Cd(II) biosorption by inactive archaea biomass is given in Table S1. Significant correlation of experimental data with the Langmuir model was shown by high *R*^2^ value (0.9072). The maximal uptake capacity (q_max_) of Cd(II) is 128.21 mg/g. The magnitude of K_f_ (sorption capacity) and n (sorption intensity) in the Freundlich isotherm indicates that metal ions may be easily separated from wastewater and significantly adsorbed^43^. As showed from the results, the n value of Cd(II) was greater than unity being 1.72. According to Sukpreabprom et al.^31^, good biosorption is demonstrated by n values between 1 and 10. The Langmuir model was determined to better fit the Cd(II) biosorption by dry archaea. This is based on the assumption of metal ions monolayer coverage across a homogenous biosorbent surface.

#### Effect of contact time and Kinetic study

Cadmium ions uptake reaches equilibrium in about 5 min of the biosorption process (Fig. 3b). The time required for heavy metals in the solution to attain a constant value is known as adsorption equilibrium time. When Cd(II) uptake was measured, it increased from 62.71% after a 5 min interval to 69.37% at 30 min. A similar result was reported by Min-Sheng et al.^36^, Polak-Berecka et al.^44^, and Sandesh et al.^45^. The rate of metal removal was initially high due to the abundance of binding sites on biomass surface which became saturated over time. The rapid Cd(II) uptake of biomass binding sites demonstrates adsorption process with no energy-mediated reactions occurred through surface binding^46^. This fast metal adsorption will make sorption application easier and more efficient^47^. Polak-Berecka et al.^44^ reported that the sorption was more effective in dry bacteria (the equilibrium time range from 3 to 10 min) than live bacteria (the equilibrium time range from 10 to 30 min). This rapid achievement of the state of equilibrium by inactivate bacteria can be explained by an increased cell surface due to bacteria disintegration after heating and thereby availability of larger numbers of functional groups. Thus, metal biosorption by inactivate archaea involved complexation between Cd(II) and a wide range of free functional groups in the current study. The rates of Cd ion sorption on dry archaeal biomass were investigated using pseudo-first- and pseudo-second-order equations (Table S2). It is noteworthy that the plot of t/qt over time (t) is linear (Fig. 3c). As seen by coefficient of determination, the Cd(II) uptake is pseudo-second-order (*R*^2^ = 0.9965) rather than pseudo first-order (*R*^2^ = 0.1646).

#### Biosorption capacity comparison between AB, BB, and IABB

The immobilization approach is a practical and cost-effective tool which can be utilized in industries. Immobilization has the advantage of allowing cells to be easily separated from the product and reused making the process economically more feasible. In the present study, non-specific physical adsorption was used since it is the simplest and easiest technique of immobilization^48^. The highest biosorption capacity was recorded for the immobilized Biomass (IABB), followed by BB and AB (Table S3).

### Surface characterization

#### SEM and EDX analysis

**Figure 4:**
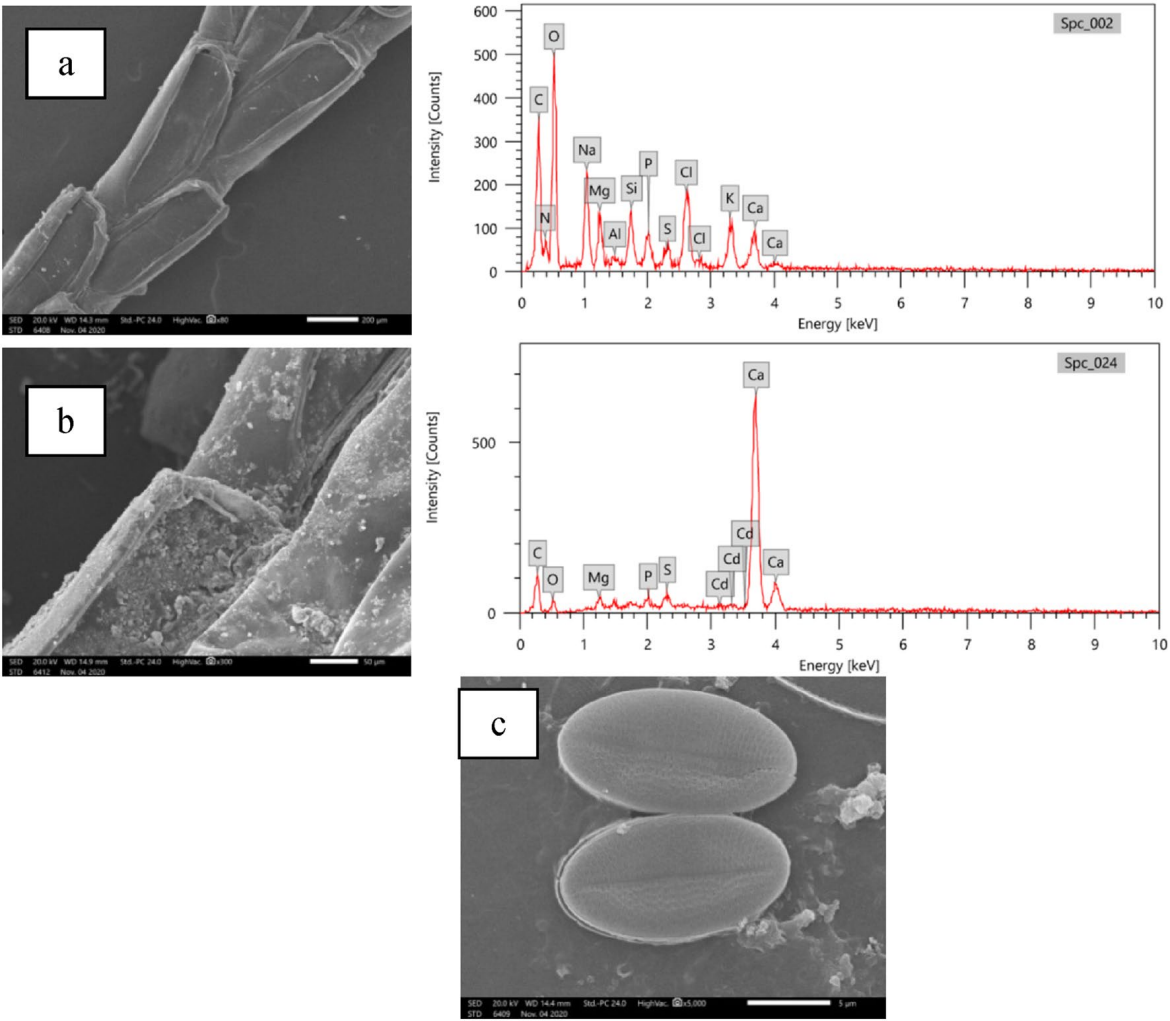
SEM images and EDX spectra of IABB, (**a**) before Cd(II) biosorption, (**b**) after Cd(II) biosorption, and (**c**) unloaded AB.

The assessment of morphological changes as a result of Cd ions accumulation on the immobilized archaeal cells has been conducted using SEM (Fig. 4). Before Cd(II) biosorption, the cells appeared to be smooth. After interaction with Cd(II), precipitates in the form of irregularly shaped aggregates appeared all over the surface of immobilised biomass (II). This result is consistent with the elemental analysis using EDX. The major elements of biomass surface were C, N, O, S, Na, K, Mg, Cl, and Ca, as shown in Fig. 4a, and Cd(II) signal was not detected in the EDX spectrum. After metal uptake, the EDX spectra of immobilised biomass showed a noticeable decrease in peaks intensity of C, O, and Mg, the disappearance of N, Na, and K peaks, and the appearance of Cd ions peaks (Fig. 4b). These findings are in accordance with that of Raize et al*.*^49^, who reported similar changes in C, O, and Mg concentrations after heavy metal biosorption by *Sargassum* sp. These alterations imply that the ion exchange process was involved in the biosorption of Cd(II) ions. A similar metal biosorption mechanism was reported by Michalak et al.^50^ and Ramyakrishna and Sudhamani^51^.

#### FT-IR analysis

FTIR spectra analyses of IABB before and after metal biosorption were conducted to obtain information regarding the possible functional groups involved in Cd(II) binding (Fig. S1). The FTIR analysis was measured in the range of 400–4000 cm^−1^. The spectra of the biosorbents displayed varied biosorption peaks indicating the composite nature of the biomass. The cell wall contains a variety of functional groups, including, carboxyl (e.g., amino acids) and hydroxyl (e.g., polysaccharides). The broadening and shifting of peaks was observed after interaction of raw biomass with the examined Cd ions. The main variations were at the wavenumber range from approximately 3200–3300 cm^−1^ (overlapping of amine N–H and hydroxyl O–H stretching vibrations), at ~ 2900 cm^−1^ (–CH stretching vibration of C–CH3), at ~ 1400–1700 cm^−1^ (stretching vibration of C = O), and at 1200–1300 cm^−1^ (bending vibrations of C-H and O–H). These changes can be attributed to the interaction between metal and organic functional groups on the cell wall.

### Heavy metal removal from multi-component system

The removal efficiencies of Cd(II), Fe(II), Pb(II), Cu(II), and Ni(II) by the halophilic AB, BB, and IABB in a multi-component polluted system were studied in this work (Table 1). Biosorption process was examined by using different levels of these heavy metals with an initial concentration of 25 mg/l (− 1) and 75 mg/l (+ 1) in the mixture system after 15 min of the experimental runs. The removal efficiency of heavy metals followed the order: Fe(II) > Pb(II) > Cd(II) > Cu(II) > Ni(II). The removal of Fe(II) and Pb(II) was in the range of ~ 67 mg/gto ~ 69 mg/g for all biomass while cadmium ions showed wide range of uptake from 64.6 mg/g for BB to 66.2 mg/g for IABB. However, a decline in the removal capacity of Cu(II) and Ni(II) were found for all experimental runs (41.4–41.8 mg/g). From the results, it was observed that the mechanism of the heavy metals removal in a multi-component polluted system was completely different from the single metal polluted system. The inhibitory effect of each metal on the other metals played a significant role in the biosorption process in multi-component system which resembles the actual wastewater-polluted system in the natural environment. Also, the initial concentration of heavy metals played an important role in metal removal in multi-component systems. In this current study, the inhibitory effect increased with the increase in heavy metals concentration. On the other hand, small ionic radius cations are assumed to transfer to adsorption sites more rapidly, but high ionic radius cations may produce an immediate saturation of the adsorbent due to steric effects, resulting in less adsorption on the adsorbent surface. In addition, the ion with the larger hydrated radius offers low forces of attraction and has greater difficulties in reaching adsorption sites^52^. Reverse trend was seen in the current investigation. However, Fe(II) biosorption may well be described by its strong affinity with the biosorbents surface functional groups. Also, the non-specific binding sites for Fe(II) present on the surface of tested biosorbents lead to its negative effect on other heavy metals removal in the multi-component system. Generally, the tested biosorbents were efficient in the removal of heavy metal, in particular Fe(II), Pb(II), and Cd(II) contaminated systems.

**Table 1:** Plackett–Burman experimental design matrix showing different combination levels of the heavy metals in the evaluation of the removal of heavy metals (mg/g) by AB, BB, and IABB.

Trials	Tested metals coded values					Removal capacity (mg/g)														
						AB					BB					IABB				
	Pb	Ni	Cu	Fe	Cd	Pb	Ni	Cu	Fe	Cd	Pb	Ni	Cu	Fe	Cd	Pb	Ni	Cu	Fe	Cd
1	− 1	1	1	− 1	1	13.59	1.65	39	0	20.75	13.59	34.69	34.385	0	46.8	13.59	40.575	41.46	0	66.275
2	− 1	− 1	1	1	− 1	14.105	9.62	22.7	68.99	0	13.59	10.23	31.04	38.435	8.675	13.59	11.2	32.905	62.99	0
3	1	− 1	− 1	1	1	37.98	16.525	8.09	0	34.42	59.6	17.135	12.54	48.535	64.63	38.865	21.14	9.435	58.39	62.825
4	1	1	− 1	− 1	1	64.38	36.575	12.66	0	18.2	63.59	38.63	15.055	12.99	28.2	65.155	48.385	12.86	0	32.865
5	− 1	1	1	− 1	− 1	13.59	31.63	37.95	14.39	17.005	13.59	29.355	40.24	12.99	15.745	13.59	35.45	38.525	0	0
6	1	− 1	1	1	− 1	67.825	12.215	27.06	62.99	1.565	63.59	10.945	26.365	62.99	15.65	63.59	8.365	29.09	46.25	14.155
7	− 1	1	− 1	1	1	12.685	37.215	7.675	39.1	35.535	13.59	38.73	12.895	60.155	63.72	13.59	41.825	8.8	46.61	62.62
8	1	− 1	− 1	− 1	− 1	63.59	11.46	37.98	0	21.95	63.59	12.535	42.25	0	27.8	63.59	7.985	37.465	0	24.4

#### Statistical analysis

**Figure 5:**
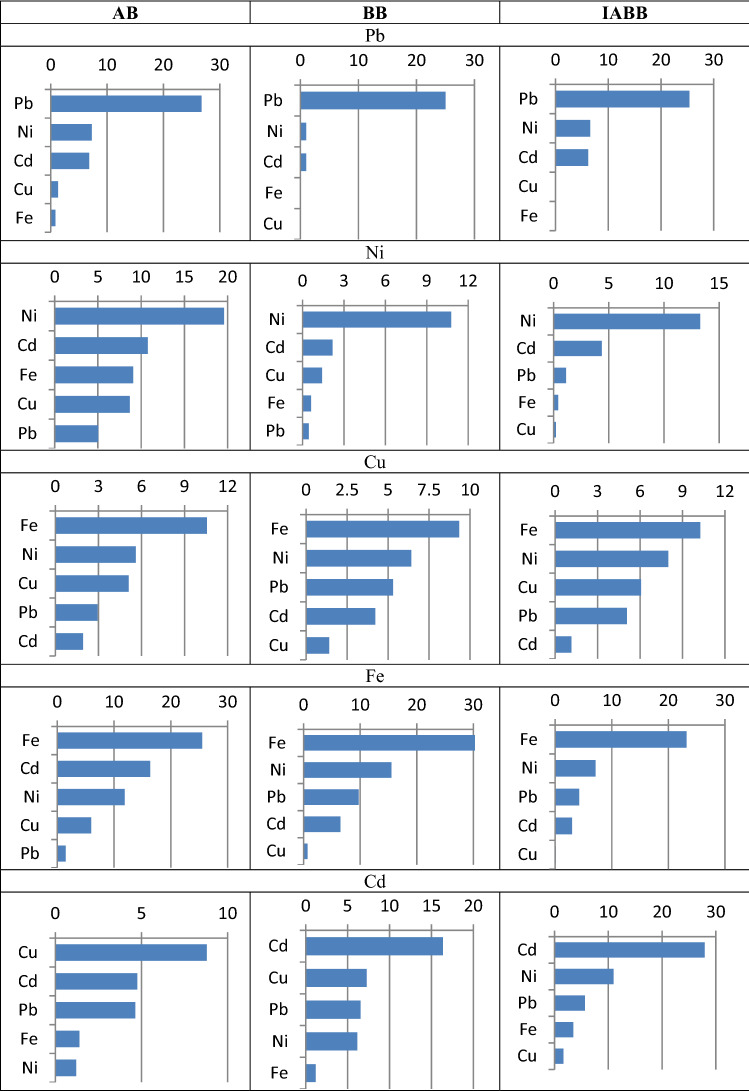
Pareto chart showing the effect of different heavy metals on each other’s removal by biosorbent.

The data of heavy metals removal obtained from the polluted aqueous solution were statistically analyzed by (ANOVA). The significance of heavy metals removal was evaluated by ANOVA, and the results are presented in Table S4 with main effect and *P* values. The deviation in the final results can be well illustrated by high* R*^2^ and low *P* value which indicated the accuracy of the efficiency of multi-component system and a great degree of correlation between the experimental and the predicted results. Also all values of the main effect, *P* value, and *R*^2^ together indicated the significance of the levels means. It was found that the effect of individual metals on their own removal and the removal of other metals in the aqueous multi-component system was analytically significant (Table S4). For example, metals Pb(II), Ni(II), Cu(II), and Fe(II) show significant positive effect on their removal (%) by all biosorbents with *P* value (0.003–0.3). While, none of the tested biosorbents showed a significant positive effect on cadmium Cd(II) metal removal (%) except IABB with *P* value 0.35. Cd(II) led to decrease in the removal of all tested metals by all tested biosorbents except for Ni in case of BB and IABB and Fe(II) in case of IABB. No effects were observed for Fe(II) and Cu(II) on Pb(II) removal in BB and IABB. In addition, Cu(II) had inhibitory effect in the removal of Ni(II) with *P* values ranged from 0.09 to 0.93. Also we found that all tested metals had inhibitory effect on the removal of Cu(II) by all biosorbents. Pb(II), Ni(II), and Cu(II) played negative role in the removal of Fe(II) in case of IABB and positive effect in case of AB and BB. Also, Pb(II), Ni(II), and Cu(II) had inhibitory effect on Cd(II) removal in all biosorbents and Fe(II) in case of AB and IABB. The inhibitory nature of some metal ions in the mixtures may be due to the screening action produced by the metals in the solution^53, 54^. The Pareto chart is described in Fig. 5. The horizontal bars in the Pareto chart show the effect of each metal in the multi-component system by all three biosorbents.

### Analysis of fixed bed column data

**Figure 6:**
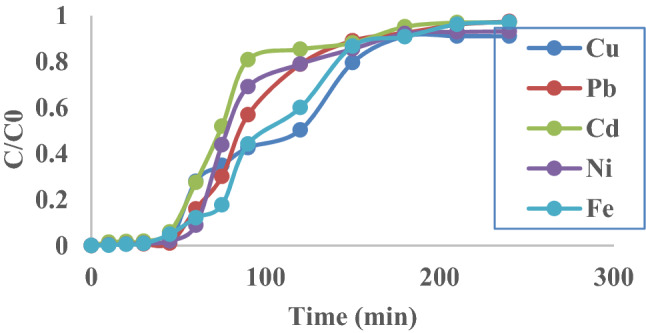
The breakthrough curve for the adsorption of simulated wastewater contaminated with Cu, Fe, Cd, Ni and Pb (flow rate of the solution is 6 ml/min; bed depth 2 cm).

To determine the life time of the adsorptive material used as a bed and the regeneration time for that bed, the breakthrough curves should be studied. Break through curve is portrayal of the concentration of pollutant-effluent versus time. When the C_0_/C value reaches 1, it means that the adsorptive material is exhausted and no more adsorption occurs. The shape of the breakthrough curve depends on the wastewater inlet flow rates, bed height, pollutants concentration and column diameter. In most cases the shape of the break through curve has the ‘S’ shape but with varying degree of gradients^55, 56^. Representation of the breakthrough curves for heavy metals under study at constant flow rate of 6 ml/min and bed height of 2 cm are shown in Fig. 6. It is observed that time of breakthrough and time of exhaustion are different for each metal. Table 2 represents the time required to break through and the time required by the adsorptive material to be exhausted with the metal.

**Table 2:** Fixed-bed column parameters for heavy metal adsorption by IABB.

Metal	Initial concentration (mg/l)	Q (ml/min)	Bed height (cm)	Mass of adsorbent (g)	Time to break through (min)	Time to exhaust (min)
Cu	4.392	6	2	1	55	180
Pb	6.835	6	2	1	65	145
Cd	4.413	6	2	1	48	110
Ni	4.532	6	2	1	60	155
Fe	5.761	6	2	1	60	200

**Figure 7:**
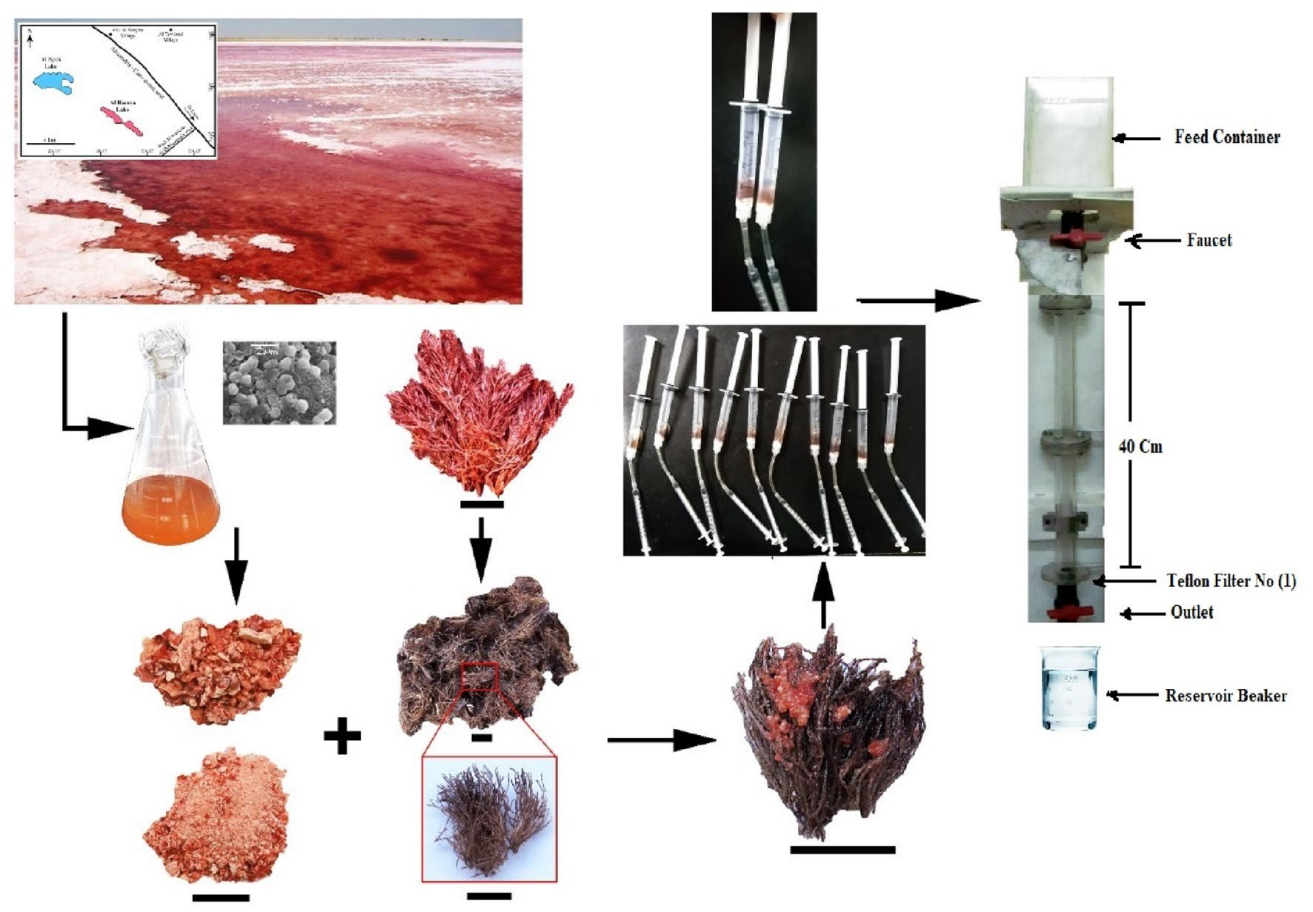
The overall biosorption process of heavy metals from wastewater.

For the Thomas model and Yoon–Nelson values, the regression coefficient *R*^2^ of 0.96 for Cu, 0.95 for Pb, ranges from 0.94 to 0.95 for Cd, 0.89 for Ni, and 0.92 for Fe, giving a superior fit for the data for both models (Table 3). Table S5 lists previous investigations on adsorption capacity of various substances for heavy metals removal. Figure 7 shows the overall biosorption process of heavy metals from wastewater.

**Table 3:** Parameters of Thomas and Yoon-Nelson models for fixed bed column adsorption process.

Metal	Q (ml/min)	Bed Height (cm)	Thomas			Yoon–Nelson		
			K_TH_*10^−4^ (L/min mg)	q_e_ (mg/g)	*R*^2^	K_YN_ (min^−1^)	Τ (min.)	*R*^2^
Cu	6	2	51.68	2838.12	0.96	0.0227	107.7	0.96
Pb	6	2	45.94	4212.01	0.95	0.0314	86.2	0.95
Cd	6	2	58.24	1987.91	0.94	0.0262	75.0	0.95
Ni	6	2	59.36	2736.59	0.89	0.0269	100.6	0.89
Fe	6	2	69.26	4222.68	0.92	0.0399	122.2	0.92

## Conclusion

Dry haloalkaliphilic archaea *Natronolimnobius innermongolicus* (AB) was evaluated for Cd(II) biosorption in Cd polluted water by using different pH, biosorbent dose, cadmium concentrations and contact time. The effect of multi-component system on the biosorption capacity of heavy metals polluted water by different types of biomass (IABB, AB, and BB) was studied using statistical Plackett–Burman experimental design. The results revealed that removal capacity was significantly influenced by the initial concentrations of heavy metals. The highest biosorption capacity was recorded for the immobilized biomass (IABB), followed by BB and AB. The removal efficiency of heavy metals followed the order: Fe(II) > Pb(II) > Cd(II) > Cu(II) > Ni(II) for all biomass. SEM, EDX, and FTIR spectra indicated changes in biomass surface characteristics during the biosorption process, which can be related to metal and functional group interactions on the cell wall of IABB.

## Supplementary Information

Supplementary Information.

## Data Availability

All data analyzed during this study are included in this published article and supplementary material.
