# Supplementary material for: Isotherm and kinetic studies of cadmium biosorption and its adsorption behaviour in multi-metals solution using dead and immobilized archaeal cells
Source: Sci Rep. 2023 Feb 13;13:2550. doi: 10.1038/s41598-023-29456-5 (PMC9925725; doi:10.1038/s41598-023-29456-5)
Supplement: Supplementary file 1 — Supplementary Information. [file 41598_2023_29456_MOESM1_ESM.docx]

**Isotherm and kinetic studies of cadmium biosorption and its adsorption behaviour in multi-metals solution using dead and immobilized archaeal cells**

Ghada E. Hegazy^1,2^, Nadia A. Soliman^2^, Mona E. Ossman^3^, Yasser R. Abdel-Fattah^2^, Madelyn N. Moawad^1^*

^1^ National Institute of Oceanography and Fisheries, NIOF, Cairo, Egypt

**^2^** Bioprocess Development Department, Genetic Engineering and Biotechnology Research Institute (GEBRI), City of Scientific Research and Technological Applications (SRTA-City), New Borg Elarab City, Alexandria, Egypt

^3^ Environment and Natural Material Research Institute (ENMRI), City for Scientific Research and Technological Applications (SRTA-City), New Borg Elarab City, Alexandria, Egypt

***Corresponding author email:** [madelynattia@gmail.com](mailto:madelynattia@gmail.com), **Tel:** +201281695012

***Corresponding author email:** [ghada19832006@yahoo.com](mailto:ghada19832006@yahoo.com), **Tel:** +201221712123

b

a

**Fig. S1** FTIR spectra of IABB biomass, a) before Cd (II) biosorption and b) after Cd (II) biosorption

**Table S1** Isotherm parameters for biosorption process of Cd ions using dry archaea biomass

| Isotherms | Parameters | | |
| --- | --- | --- | --- |
| Langmuir model | q_max_ (mg/g) | b (l/mg) | *R^2^* |
|  | 128.21 | 0.05 | 0.9072 |
| Freundlich model | N | K_f_ | *R^2^* |
|  | 1.72 | 9.62 | 0.7886 |

**Table S2** kinetic parameters for biosorption process of Cd ions using dry archaea biomass

| Kinetic model | q_e(exp_._)_ (mg/g) | q_e(thero_._)_  (mg/g) | K (min^-1^) | *R^2^* |
| --- | --- | --- | --- | --- |
| First order equation | 69.37 | 3.12 | 0.06 | 0.1646 |
| Kinetic model | q_e(exp.)_  (mg/g) | q_e(thero.)_  (mg/g) | K (g/mg.min) | *R^2^* |
| Second order equation | 69.37 | 67.57 | 0.07 | 0.9965 |

**Table S3** Comparison of biosorption capacity for AB, BB, and IABB

| Biomass | q_e_ (mg/g) | % Removal |
| --- | --- | --- |
| AB | 4.09 | 58.78 |
| BB | 5.27 | 75.88 |
| IABB | 6.31 | 90.79 |

**Table S4** Statistical analysis of Plackett-Burman Design experiment for heavy metals removal by AB, BB and IABB

| Name | AB | | | | | | | BB | | | | | | | | IABB | | | | | | |
| --- | --- | --- | --- | --- | --- | --- | --- | --- | --- | --- | --- | --- | --- | --- | --- | --- | --- | --- | --- | --- | --- | --- |
|  |  | | | | | | |  | | | | | | | |  | | | | | | |
|  | *Coefficients* | | *t Stat* | | | *P-value* | | *Coefficients* | | | *t Stat* | | | *P-value* | | *Coefficients* | | *t Stat* | | | *P-value* | |
| For Pb(II) | | | | | | | | | | | | | | | | | | | | | | |
| Intercept | 35.96813 | | 8.651241 | | | 0.013099 | | 38.0912 | | | 62.35865 | | | 0.000257 | | 35.695 | | 9.21498 | | | 0.011561 | |
| Pb | 26.77 | | 4.072293 | | | 0.055342 | | 25 | | | 25.8846 | | | 0.001489 | | 25.39125 | | 4.147763 | | | 0.053504 | |
| Ni | 7.30375 | | 0.878368 | | | 0.472386 | | 0.9975 | | | 0.816497 | | | 0.5 | | 6.5725 | | 0.84879 | | | 0.485387 | |
| Cu | 1.285 | | 0.218549 | | | 0.847275 | | 0 | | | 0 | | | 1 | | 0 | | 0 | | | 1 | |
| Fe | 0.8325 | | 0.141589 | | | 0.900379 | | 0 | | | 0 | | | 1 | | 0 | | 0 | | | 1 | |
| Cd | -6.81875 | | -1.03728 | | | 0.408566 | | -0.9975 | | | -1.0328 | | | 0.410232 | | -6.18125 | | -1.00973 | | | 0.418922 | |
| For Ni(II) | | | | | | | | | | | | | | | | | | | | | | |
| Intercept | 19.61125 | | 6.948707 | | | 0.020089 | | 24.03063 | | | 73.66621 | | | 0.000184 | | 26.86563 | | 16.94977 | | | 0.003463 | |
| Pb | 5.02875 | | 1.126909 | | | 0.376811 | | 0.460625 | | | 0.89306 | | | 0.466061 | | 1.135 | | 0.45289 | | | 0.695016 | |
| Ni | 19.595 | | 3.471475 | | | 0.073899 | | 10.76875 | | | 16.50588 | | | 0.00365 | | 13.28125 | | 4.18963 | | | 0.052522 | |
| Cu | -8.7025 | | -2.18036 | | | 0.161026 | | -1.40875 | | | -3.05367 | | | 0.092587 | | -0.2175 | | -0.09703 | | | 0.93155 | |
| Fe | 9.08 | | 2.274938 | | | 0.150725 | | 0.61375 | | | 1.330392 | | | 0.314808 | | 0.4075 | | 0.181794 | | | 0.872501 | |
| Cd | -10.7688 | | -2.4132 | | | 0.137236 | | 2.175625 | | | 4.218104 | | | 0.05187 | | 4.36625 | | 1.742229 | | | 0.223592 | |
| For Cu(II) | | | | | | | | | | | | | | | | | | | | | | |
| Intercept | 24.13938 | | 8.063541 | | | 0.015034 | | 26.84625 | | | 15.60038 | | | 0.004084 | | 26.3175 | | 12.78015 | | | 0.006067 | |
| Pb | -2.94438 | | -0.62205 | | | 0.597374 | | -5.30063 | | | -1.94809 | | | 0.190755 | | -.06813 | | -1.55657 | | | 0.259859 | |
| Ni | -5.60625 | | -0.93636 | | | 0.447935 | | -6.415 | | | -1.86388 | | | 0.203357 | | -7.9975 | | -1.94185 | | | 0.191652 | |
| Cu | 5.10125 | | 1.204929 | | | 0.351462 | | 1.40125 | | | 0.575774 | | | 0.62292 | | 6.07125 | | 2.084753 | | | 0.172443 | |
| Fe | -10.5613 | | -2.4946 | | | 0.130069 | | -9.34375 | | | -3.83935 | | | 0.061634 | | -0.2588 | | -3.52266 | | | 0.071992 | |
| Cd | -1.92938 | | -0.40761 | | | 0.723049 | | -4.21938 | | | -1.55071 | | | 0.261123 | | -.14438 | | -0.35147 | | | 0.758809 | |
| For Fe(II) | | | | | | | | | | | | | | | | | | | | | | |
| Intercept | 23.18375 | | 3.301353 | | | 0.080789 | | 29.51188 | | | 19.02637 | | | 0.002751 | | 26.78 | | 10.35743 | | | 0.009193 | |
| Pb | 1.48625 | | 0.133854 | | | 0.905772 | | 9.740625 | | | 3.971696 | | | 0.057939 | | -4.23 | | -1.03469 | | | 0.409526 | |
| Ni | 11.8725 | | 0.845319 | | | 0.486937 | | 15.53875 | | | 5.008934 | | | 0.037623 | | -7.13 | | -1.3788 | | | 0.301915 | |
| Cu | 5.9725 | | 0.601381 | | | 0.608672 | | 0.70875 | | | 0.3231 | | | 0.777273 | | -0.09 | | -0.02461 | | | 0.982598 | |
| Fe | 25.5225 | | 2.569902 | | | 0.123895 | | 30.78625 | | | 14.03463 | | | 0.005039 | | 23.215 | | 6.348853 | | | 0.023922 | |
| Cd | -16.3588 | | -1.47329 | | | 0.278577 | | -6.50688 | | | -2.65315 | | | 0.117537 | | 2.99 | | 0.731379 | | | 0.540632 | |
| For Cd(II) | | | | | | | | | | | | | | | | | | | | | | |
| Intercept | 18.67813 | | 4.395488 | | | 0.048058 | | 33.9025 | | | 5.353737 | | | 0.033163 | | 32.8925 | | 4.465551 | | | 0.046665 | |
| Pb | -4.6425 | | -0.69096 | | | 0.56101 | | -6.54 | | | -0.65318 | | | 0.580695 | | -5.6375 | | -0.48406 | | | 0.676165 | |
| Ni | -1.20375 | | -0.14164 | | | 0.900345 | | -6.1475 | | | -0.48539 | | | 0.675364 | | -10.965 | | -0.74432 | | | 0.534257 | |
| Cu | -8.7925 | | -1.46309 | | | 0.280985 | | -7.2675 | | | -0.81151 | | | 0.502294 | | -1.6475 | | -0.15816 | | | 0.888859 | |
| Fe | -1.4 | | -0.23296 | | | 0.837461 | | 1.1925 | | | 0.133158 | | | 0.906257 | | -3.475 | | -0.33359 | | | 0.770414 | |
| Cd | 4.75375 | | 0.707522 | | | 0.552576 | | 16.375 | | | 1.635448 | | | 0.243585 | | 27.9125 | | 2.396663 | | | 0.138759 | |
| ANOVA | | | | | | | | | | | | | | | | | | | | | | |
| Biomass | AB | | | | | | | | BB | | | | | | | IABB | | | | | | |
|  | *dd* | *ss* | | *MS* | *F* | | SigniF | | *dd* | *ss* | | *MS* | *F* | | SignF | *dd* | *ss* | | *MS* | *F* | | SigniF |
| For Pb(II) | | | | | | | | | | | | | | | | | | | | | | |
| Regression | 5 | 4334.206 | | 866.84 | 6.26 | | 0.14327 | | 5 | 4808.46 | | 961.69 | 2.985 | | 0.003097 | 5 | 4148.887 | | 829.7775 | 6.919448 | | 0.1310 |
| Residual | 2 | 276.566 | | 138.283 |  | |  | | 2 | 5.970038 | | 322.173 |  | |  | 2 | 239.8392 | | 119.9196 |  | |  |
| Total | 7 | 4610.772 | |  |  | |  | | 7 | 4814.43 | |  |  | |  | 7 | 4388.727 | |  |  | |  |
| *R^2^* | 0.94001 |  | |  |  | |  | | 0.9987 |  | |  |  | |  | 0.945351 |  | |  |  | |  |
| For Ni(II) | | | | | | | | | | | | | | | | | | | | | | |
| Regression | 5 | 1167.77 | | 233.5 | 3.66 | | 0.22814 | | 5 | 1110.765 | | 222.1531 | 260.957 | | 0.0038 | 5 | 1885.2 | | 377.0508 | 18.7604 | | 0.05137 |
| Residual | 2 | 124.4 | | 63.7 |  | |  | | 2 | 1.702603 | | 0.851302 |  | |  | 2 | 40.196 | | 20.09817 |  | |  |
| Total | 7 | 1295.2 | |  |  | |  | | 7 | 1112.468 | |  |  | |  | 7 | 1925.4 | |  |  | |  |
| *R^2^* | 0.902 |  | |  |  | |  | | 0.9923 |  | |  |  | |  | 0.9791 |  | |  |  | |  |
| For Cu(II) | | | | | | | | | | | | | | | | | | | | | | |
| Regression | 5 | 1130.78 | | 226.1 | 3.154 | | 0.25804 | | 5 | 982.2773 | | 196.4555 | 8.29 | | 0.111 | 5 | 1258.805 | | 251.761 | 7.42 | | 0.122994 |
| Residual | 2 | 143.39 | | 71.6952 |  | |  | | 2 | 47.38236 | | 23.691 |  | |  | 2 | 67.84802 | | 33.92 |  | |  |
| Total | 7 | 1274.17 | |  |  | |  | | 7 | 1029.66 | |  |  | |  | 7 | 1326.6 | |  |  | |  |
| *R^2^* | 0.887464 |  | |  |  | |  | | 0.9539 |  | |  |  | |  | 0.948858 |  | |  |  | |  |
| For Fe(II) | | | | | | | | | | | | | | | | | | | | | | |
| Regression | 5 | 5374.304 | | 1074.86 | 2.724 | | 0.28999 | | 5 | 4750.63 | | 950.1278 | 49.36401 | | 0.01997 | 5 | 5844.376 | | 1168.8 | 21.85553 | | 0.04432 |
| Residual | 2 | 789.048 | | 394.524 |  | |  | | 2 | 38.4947 | | 19.24738 |  | |  | 2 | 106.96 | | 53.48 |  | |  |
| Total | 7 | 6163.352 | |  |  | |  | | 7 | 4789.13 | |  |  | |  | 7 | 5951.3 | |  |  | |  |
| *R^2^* | 0.871977 |  | |  |  | |  | | 0.9919 |  | |  |  | |  | 0.98202 |  | |  |  | |  |
| For Cd(II) | | | | | | | | | | | | | | | | | | | | | | |
| Regression | 5 | 902.8016 | | 180.560 | 1.249914 | | 0.50048 | | 5 | 2727.035 | | 545.40 | 1.700127 | | 0.410356 | 5 | 4613.033 | | 922.6066 | 2.125607 | | 0.35018 |
| Residual | 2 | 288.9165 | | 144.458 |  | |  | | 2 | 641.6073 | | 320.80 |  | |  | 2 | 868.0876 | | 434.0438 |  | |  |
| Total | 7 | 1191.718 | |  |  | |  | | 7 | 3368.642 | |  |  | |  | 7 | 5481.12 | |  |  | |  |
| *R^2^* | 0.75756 |  | |  |  | |  | | 0.8095 |  | |  |  | |  | 0.8416 |  | |  |  | |  |

**Table S5** Comparison between adsorption capacities of IABB and some adsorbents for heavy metal uptakes in fixed-bed columnn

| Adsorptive material | Metal | Conc. (mg/l) | Flow rate (ml/min) | Bed height (cm) | Adsorption capacity (mg/g) | τ  (min) | Ref. |
| --- | --- | --- | --- | --- | --- | --- | --- |
| Charcoal Originated from Chemical Carbonization of Rubber Wood Sawdust | Pb | 10 | 15 | 5 | 36884.55 | 624.5 | Biswas and Mishra (2015) |
| Activated charcoal from Neem (*Azadirachtaindic*) leaf powder | Pb | 25 | 5 | 10 | 25000 | 93 | Patel (2020) |
|  | Cr | 25 | 5 | 10 | 8717.11 | 61 |  |
| Chitosan-Coated Bentonite | Pb | 50 | 0.4 | 2 | 11.53 | 1370 | Tsai et al. (2016) |
|  | Cu | 50 | 0.4 | 2 | 10.12 | 1181.8 |  |
|  | Ni | 50 | 0.4 | 2 | 9.09 | 948.8 |  |
| Activated hydrochar | Cd | 10 | 12 | 1.3 | 4.19 | 12 | (Rind et al., 2022) |
| Cockle Shell (*Anadara Granosa*) Powder | Cd | 190 | 8 | 4.3 | 157.51 | 484.09 | (Nguyena et al., 2021) |
| IABB | Cu | 4.392 | 6 | 2 | 2838.12 | 107.7 | Current study |
|  | Pb | 6.835 | 6 | 2 | 4212.01 | 86.2 |  |
|  | Cd | 4.413 | 6 | 2 | 1987.91 | 75.0 |  |
|  | Ni | 4.532 | 6 | 2 | 2736.59 | 100.6 |  |
|  | Fe | 5.761 | 6 | 2 | 4222.68 | 122.2 |  |

**References**

Biswas, S., Mishra, U., 2015. Continuous Fixed-Bed Column Study and Adsorption Modeling: Removal of Lead Ion from Aqueous Solution by Charcoal Originated from Chemical Carbonization of Rubber Wood Sawdust. J. Chem. 2015, 907379.

Nguyena, T.-A., Nhan, C.-H., Le, M.-V., Huynh, P.-H.K., Phung, T.K., Tran, A.V., 2021. Fixed Bed Column Studies for the Adsorption of Cadmium onto Cockle Shell (Anadara Granosa) Powder. Chem. Eng. Trans. 83, 259–264. https://doi.org/10.3303/CET2183044

Patel, H., 2020. Batch and continuous fxed bed adsorption of heavy metals removal using activated charcoal from neem (Azadirachtaindica) leaf powder. Sci. Rep. 10, 16895.

Rind, I.K., Memon, N., Khuhawar, M.Y., Soomro, W.A., Lanjwani, M.F., 2022. Modeling of cadmium(II) removal in a fixed bed column utilizing hydrochar-derived activated carbon obtained from discarded mango peels. Sci. Rep. 12, 8001. https://doi.org/10.1038/s41598-022-11574-1

Tsai, W.C., de Luna, M.D., Bermillo-Arriesgado, H.L.P., Futalan, C.M., Colades, J.I., Wan, M.W., 2016. Competitive Fixed-Bed Adsorption of Pb (II), Cu (II), and Ni (II) from Aqueous Solution Using Chitosan-Coated Bentonite. Int. J. Polym. Sci. 2016, 1608939.
